# Supplementary material for: The Link between ADHD Symptoms and Antisocial Behavior: The Moderating Role of the Protective Factor Sense of Coherence
Source: Brain Sci. 2022 Oct 3;12(10):1336. doi: 10.3390/brainsci12101336 (PMC9599088; doi:10.3390/brainsci12101336)
Supplement: Supplementary file 1 [file brainsci-12-01336-s001.zip › brainsci-1916537-supplementary.pdf]

Supplementary:

Table S1- Nonparametric Correlations between demographic characteristics, ADHD symptoms, antisocial behavior types and coherence.

	Age	Gender	Religiosity	Emotional symptoms
**ADHD symptoms**	-.123**	-0.008	-.148**	.343**
**Severe physical antisocial behavior**	-.252**	-.087**	-.044**	.065**
**Mild physical antisocial behavior**	-.240**	-.109**	-0.008	.101**
**Verbal antisocial behavior**	-.233**	-.080**	-.157**	.173**
**Property crimes**	-.210**	-.104**	-.144**	.136**
**Public order crimes**	-.246**	-.059**	-.178**	.100**
**Drug use**	.053	-.348**	-.231**	-0.004
**Coherence**	.208**	-.099**	.290**	-.606**

**. Correlation is significant at the 0.01 level (2-tailed).

Table S2- Pearson correlations between the research variables

	1	2	3	4	5	6	7	8
**1. ADHD symptoms**	1							
**2. Severe physical antisocial behavior**	.220**	1						
**3. Mild physical antisocial behavior**	.233**	.694**	1					
**4. Verbal antisocial behavior**	.249**	.451**	.574**	1				
**5. Property crimes**	.173**	.655**	.574**	.382**	1			
**6. Public order crimes**	.269**	.453**	.489**	.543**	.443**	1		
**7. Drug use**	.127**	.286**	.265**	.270**	.300**	.362**	1	
**8. Coherence**	-.424**	-.215**	-.307**	-.380**	-.229**	-.309**	-.129**	1

**. Correlation is significant at the 0.01 level (2-tailed).
